# Cerebral Outflow Discrepancies in Recurrent Benign Paroxysmal Positional Vertigo: Focus on Ultrasonographic Examination

**DOI:** 10.3390/diagnostics13111902

**Published:** 2023-05-29

**Authors:** Andrea Ciorba, Mirko Tessari, Erennio Natale, Fabio Buzzi, Giulia Baldazzi, Alessio Cosacco, Andrea Migliorelli, Virginia Corazzi, Chiara Bianchini, Francesco Stomeo, Stefano Pelucchi, Paolo Zamboni

**Affiliations:** 1 ENT & Audiology Unit, Department of Neurosciences, University Hospital of Ferrara, 44124 Ferrara, Italy; 2 Vascular Diseases Centre, University Hospital of Ferrara, 44124 Ferrara, Italy

**Keywords:** benign paroxysmal positional vertigo, labyrinth diseases, chronic cerebrospinal venous insufficiency, jugular veins, ultrasonography doppler

## Abstract

This prospective pilot study aimed to evaluate whether cerebral inflow and outflow abnormalities assessed by ultrasonographic examination could be associated with recurrent benign paroxysmal positional vertigo (BPPV). Twenty-four patients with recurrent BPPV, affected by at least two episodes, and diagnosed according to American Academy of Otolaryngology–Head and Neck Surgery (AAO–HNS) criteria, evaluated at our University Hospital, between 1 February 2020 and 30 November 2021, have been included. At the ultrasonographic examination, 22 of 24 patients (92%) reported one or more alterations of the extracranial venous circulation, among those considered for the diagnosis of chronic cerebrospinal venous insufficiency (CCSVI), although none of the studied patients were found to have alterations in the arterial circulation. The present study confirms the presence of alterations of the extracranial venous circulation in recurrent BPPV; these anomalies (such as stenosis, blockages or regurgitation of flow, or abnormal valves, as per the CCSVI) could cause a disruption in the venous inner ear drainage, hampering the inner ear microcirculation and then possibly causing recurrent otolith detachment.

## 1. Introduction

Benign paroxysmal positional vertigo (BPPV) is one of the most common forms of peripheral vestibular pathologies, accounting for approximately 20–40% of all diagnoses [1]. The impact on the quality of life of undiagnosed (and therefore untreated) BPPV can be very disabling, particularly in its recurrent form, as these patients can have an increased risk of falls and impairment in their daily activities [2]. The prevalence of this condition in the general population stands at 10–64 per 100,000, with an average prevalence of 2.4% and an annual incidence of 1.6%, according to the literature [3]. Females are twice as likely as men to be affected, at an average age of >40 years; furthermore, BPPV mainly involves the right posterior semicircular canal [4].

To date, little is known about BPPV etiology, and particularly its recurrent form. In fact, several conditions have been associated with recurrent BPPV, such as metabolic disorders, vestibular neuritis, autoimmune diseases, sudden sensorineural hearing loss, Ménière’s disease (MD), vitamin D deficiency, and vascular (and microvascular) disorders. Therefore, it is possible to speculate about a possible link with chronic cerebrospinal venous insufficiency (CCSVI) [5,6,7]. It is universally accepted, in the literature, that the internal jugular veins (IJVs) represent the main cerebral drainage route in the supine position, whilst the vertebral veins (VVs) are in the upright position [8]. The IJV is subdivided echographically into three segments: the J3, or superior segment, which is anatomically located at the carotid bifurcation and the mandibular angle; the middle segment, or J2, which is related to the ipsilateral thyroid lobe; and the inferior segment, or J1, which corresponds to the confluence with the brachio-cephalic venous trunk [9]. The flow tends to physiologically increase in volume from J3 to J1 [10]. In 2006, the five ultrasound parameters that indicate anatomical–functional alterations of the venous blood flow in the neck, defining CCSVI, were specifically described [11]. CCSVI is a chronic vascular condition characterized by restricted venous outflow from the brain, mainly due to narrowing or obstruction of veins of head and neck. This condition can lead to the formation of replacement circles, venous reflux, and iron deposition in the central nervous system [8,11]. In 2009, the International Union of Phlebology Consensus Document on Venous Malformations, defined CCSVI as a unique venous disease [12,13]. In the same year, an endovascular treatment of CCSVI lesions by percutaneous angioplasty was proposed [14]. Echo-color Doppler (ECD) is the gold standard procedure for measuring flow parameters. In a recent study, it was shown that, in healthy subjects, only a very small amount (1%) of blood volume in physiology re-enters through the collaterals into the caval system, skipping the IJV. In contrast, in patients with CCSVI, this percentage increases dramatically to around 60%, leading to a likely slowing of cerebral flow and increasing stasis [8].

Recently, a number of authors in the literature have investigated whether the anatomo-functional alterations of CCSVI may contribute to alterations of the inner ear. Several studies have analyzed and demonstrated a correlation between CCSVI and inner ear diseases such as MD and sudden sensorineural hearing loss (SSNHL) [15,16,17,18,19,20,21,22]. Moreover, for MD, it has recently been reported that a venous angioplasty procedure could offer positive results on symptom control in selected patients [23]. To the best of our knowledge, the present study represents the first clinical investigation of a possible correlation between CCSVI and recurrent BPPV.

The aim of this study was to evaluate, by ultrasonographic examination, the brain inflow and outflow of a cohort of patients with recurrent BPPV, eventually assessing cerebral inflow and/or outflow abnormalities.

## 2. Patients and Methods

### 2.1. Type of Study

This was a prospective pilot study.

### 2.2. Patients

The study group included a total of 24 patients with recurrent BPPV evaluated at our University Hospital between 1 February 2020 and 30 November 2021.

### 2.3. Vestibular Assessment

BPPV was diagnosed according to the American Academy of Otolaryngology–Head and Neck Surgery (AAO–HNS) criteria [24]. BPPV diagnosis was performed in the presence of vertigo associated with typical nystagmus induced by diagnostic maneuvers (Dix-Hallpike maneuver to assess posterior semicircular canal BPPV, and Pagnini–McClure maneuver or supine roll test to assess lateral semicircular canal BPPV). If the diagnostic test was positive at the time of physical examination, the patient was treated with the appropriate repositioning procedure (Epley or liberatory Semont maneuver for the posterior canal and Gufoni or Barbecue roll maneuver for the lateral canal). According to Sfakianaki et al. [25], “recurrent” BPPV is defined as a BPPV with symptoms and nystagmus recurring after at least 4 weeks of symptom-free intervals after previous successful treatment. Subjects were enrolled in the study according to the following inclusion criteria: (i) at least 2 BPPV episodes (with positive provocative maneuver and typical nystagmus) occurring at an interval of minimum 4 weeks, in which diagnostic maneuvers were negative; and (ii) age between 18 and 70 years. Exclusion criteria were: (i) peripheral vertigo not meeting the AAO–HNS diagnostic criteria for BPPV; (ii) non-peripheric (central or other) vertigo; (iii) age below 18 or over 70 years; and (iv) history of other cochleovestibular disorders. Patient medical history was also collected, including biographical data, gender, previous episodes of vertigo, diagnostic investigations performed, treatment received, and persistence of symptoms after treatment. All included patients underwent cerebral magnetic resonance imaging (MRI) to rule out retrocochlear pathologies.

### 2.4. Vascular Assessment

Ultrasonographic study of the arterial and venous circulation of the neck, with particular attention to the IJVs and the VVs, and of the intracranial venous system, was performed by the same operator. The ECD protocol described by Lugli et al. [26] was used in sampling the common carotid artery (CCA), the external and internal carotid artery (ECA and ICA, respectively), and the vertebral artery (VA). Parameters of time-averaged velocity (TAV), cross-sectional area (CSA), and flow rate were assessed according to reporting standards [27]. The assessment of the IJV outflow was accomplished by insonating the vessel at three classic levels, identified as J3 (superior, the upper part of the neck just below the jugular foramen), J2 (intermediate, at the level of the mid thyroid gland), and J1 (inferior, outlet with the subclavian vein); VVs were investigated at the V2 level (at the level of C5–C6′s transverse processes) [9]. For the venous circulation, TAV, peak velocity, CSA, and flow rate were assessed. The protocol procedure for the vascular assessment was performed as described below: a color Doppler ultrasound (US) system with a linear vascular probe (7–12 MHz) was used to evaluate the extracranial venous circulation of the neck, while an intracranial probe (2–3 MHz) was used to analyze the veins of deep cerebral circulation and the cerebral sinuses. The examination was primarily performed in the supine position, with the patient breathing spontaneously through their nose (without requiring Valsalva maneuvers). The whole process was repeated after 2–3 min with the patient in a sitting position at 90° and eyes facing to the front, after a 2–3 min wait. First, the extracranial circle was assessed, and then the intracranial circle. Evaluation of the extracranial circulation began from the right IJV, in a transverse plane, starting from the base of the neck (J1) to the mandibular angle (J3). The identified valves were evaluated in both the transverse and longitudinal planes in M-mode. Proceeding on the transverse plane, the measurement of CSA in B-mode was also accomplished starting from the J2 level (thyroid plane) in both supine and sitting positions. Then, the longitudinal plane was assessed starting from the middle portion (J2). The direction of flow was observed using color; subsequently, the direction of flow and duration of reflux or bidirectional flow were assessed through Doppler spectrum analysis. These measurements were repeated at levels J3 and then J1. VV evaluation was performed in B-mode, using ECD, with a probe placed longitudinally. The diagnosis of CCSVI was established according to the criteria of the Consensus Conference of the 2011 International Society for Neurovascular Disease (ISNVD), Annual Meeting in Bologna, Italy [9,28]. The diagnosis of CCSVI was based on the presence of at least two of the following four alterations: (i) reflux in the IJV and/or VV; (ii) valvular abnormalities of IJV resulting in stenosis; (iii) absence of flow in the IJV and/or VV; (iv) alterations in the IJV’s CSA.

### 2.5. Statistical Analysis

Data are expressed as the mean ± sd (standard deviation); differences among the hemodynamic parameters were assessed in BPPV patients. For the statistical analysis, the non-parametric Student’s *t*-test and the Wilcoxon matched-pairs signed rank test were assessed using GraphPad Prism 9.4.1, version 2022 for Windows.

### 2.6. Ethical Consideration

The study was approved by the local ethics committee of our University Hospital (Comitato Etico Area Vasta Emilia Centro, CE-AVEC), with protocol number 101,298.

The research was conducted ethically, with all study procedures being performed in accordance with the requirements of the World Medical Association’s Declaration of Helsinki.

Written informed consent was obtained from each patient for study participation and data publication.

## 3. Results

In total, 24 patients diagnosed with recurrent BPPV were included. The study group consisted of 5 men (21%) and 19 women (79%), with a male-to-female ratio of 0.26, and a mean age of 57.7 ± 7.05 years. Demographic and clinical characteristics of the cohort are shown in Table 1.

**Figure 1:**
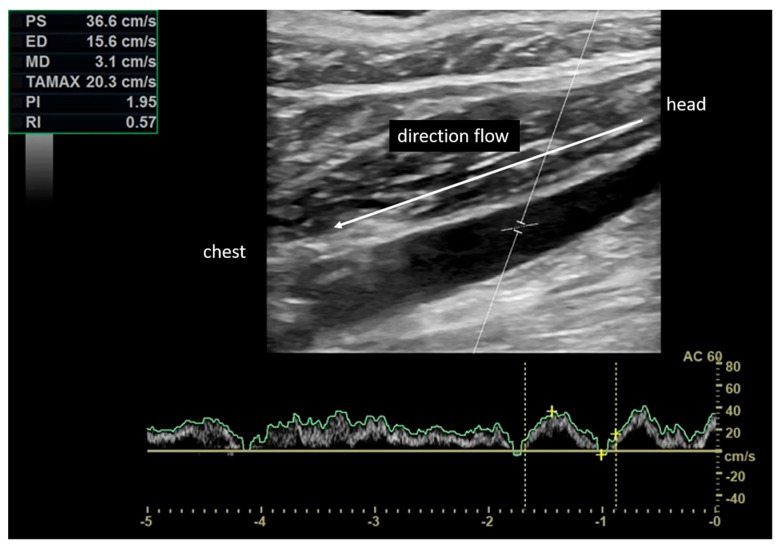
Physiological flow in the internal jugular vein normodirected and phasic with breathing. Peak velocity of 36.6 cm/s.

The mean interval between the first and the second BPPV episode was 18.1 ± 20 weeks. Of the studied patients, 83% (20 subjects) had posterior semicircular canal BPPV, while 17% (4 subjects) had lateral canal BPPV. In particular, 16 subjects (67%) were affected by BPPV of the right semicircular canal (12 affecting the posterior canal and 4 affecting the lateral canal), while 8 patients (33%) had left posterior semicircular canal BPPV. None of the patients exhibited left lateral semicircular canal involvement. Figure 1 shows a physiological flow in the normodirected and phasic IJV with breathing.

**Figure 2:**
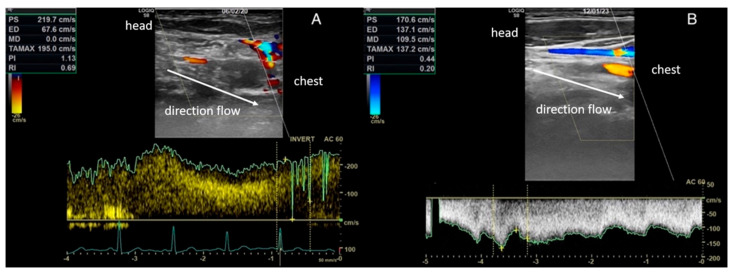
Pathological flow showing high velocity and turbulence in the internal jugular vein (peak velocities of 219.7 cm/s (**A**) and 170.6 cm/s) (**B**).

**Figure 3:**
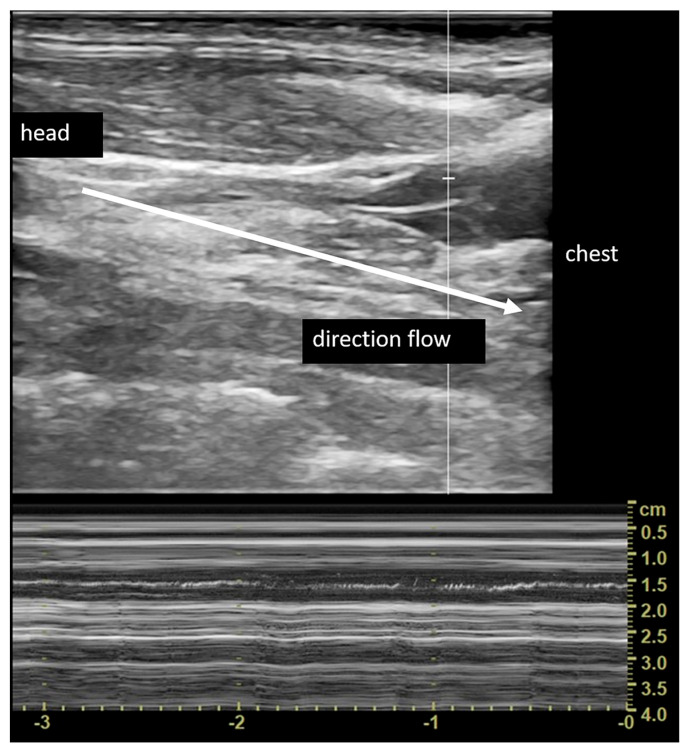
Hypomobile valvular septum obstructing the lumen of the internal jugular vein at M-mode analysis.

In total, 22 of 24 patients (92%) reported one or more alterations of the extracranial venous circulation at the US examination, among those considered for the diagnosis of CCSVI. In particular, 22/24 patients (92%) had at least one vascular alteration ipsilateral to the vestibule affected by recurrent BPPV; 6 patients (25%) had two vascular alterations (with at least one alteration ipsilateral to the affected vestibule); and 5 patients (21%) showed three vascular alterations (with at least one alteration ipsilateral to the affected vestibule). The vascular alterations diagnosed by US examination were: the absence of VV flows at least on one side in 14 patients (58%); increased IJVs flow velocity in the orthostatic position with a TAVm (time average mean velocity) from 60.0 cm/s to 126.8 cm/s (meaning an abnormal reduction in the venous lumen in the upright posture, indicating hypoplasia of the IJV) in 19 patients (79%) (Figure 2); and valvular abnormalities (fixed and hypomobilized flaps) at the J1 level, resulting in IJV flow abnormality/blockage in 5 patients (21%) (Figure 3). None of the studied patients were found to have alterations in the arterial circulation.

The hemodynamic values are reported in Table 2.

## 4. Discussion

Vertigo represents, after headaches, the second most frequent symptom reported by the general population; it accounts for 8% of all cases seeking the attention of a general practitioner and about 3.3% of all visits to the Emergency Room, according to the literature [29,30].

BPPV is among the most frequent peripheral vestibular disorders [1]. Its prevalence in the general population is estimated to be around 10%, and it has been reported to account for 20–40% of all dizziness cases [1,31]. BPPV, especially when recurrent, can have important implications in terms of quality-of-life perception, and hence, a significant economic and social impact [2,31,32,33,34,35,36,37,38,39,40].

According to the literature, BPPV is more frequent in females, and, also considering a recent meta-analysis, women are at higher risk of BPPV recurrence, as per the data reported here [32]. Advanced age is a further risk factor for the development of recurrent BPPV; Piccioti et al. [33] showed that the risk of recurrence of BPPV in patients older than 65 years is 1.6 times higher than in those younger than 65 years.

Moreover, according to the results of this series, the interval between the first and the second BPPV episode is approximately 5 months, on average. In the literature, it has been reported that in 50% of cases, recurrent BPPV episodes occur within 6 months, and it has also been observed that, in general, about 27% of patients affected by BPPV can suffer further episodes [34].

However, little is known about BPPV etiology, specifically concerning the detachment mechanisms of otoliths from the utricle macula [25]. In fact, in more than 70% of cases, the BPPV etiology remains unknown [41], and even more scant is the information available among the cause of recurrent forms. Head trauma is reported to be the most known etiology, accounting for approximately 8.5–20% of all cases [42]. In addition, an association between BPPV and vitamin D deficiency has recently been proposed; patients with recurrent BPPV have significantly lower levels of vitamin D, and its supplementation can eventually reduce recurrences [43].

However, many studies have suggested that BPPV, especially in its recurrent form, can be related to vascular disorders, and particularly to damage of the inner ear microcirculation. In fact, patients with cardiovascular risk factors, such as hyperlipidemia, hypertension, and diabetes mellitus show higher rates of recurrent BPPV [25]. This has been linked to a distressed inner ear microcirculation and to a consequent induced oxidative stress, at the inner ear macular and cellular level, causing otolith detachment [44]. According to Zhang et al. [45], the presence of stenosis of the vertebral or basilar arteries could correlate to the onset of BPPV. Furthermore, Neri et al. [46] hypothesized that a chronic ischemic reduction in vertebral flow could induce degeneration of the utricular macula, therefore contributing to the development of recurrent BPPV.

The present study investigates the hypothesis that, in addition to inflow impairments, brain outflow chronic abnormalities may cause inner ear chronic disorders, including recurrent BPPV, promoting utricular macular damage and then recurrent otolith detachment. In fact, it has already been reported that a condition of chronic outflow abnormality can possibly corelate to the onset of other audio-vestibular disorders, such as MD and SSNHL [15,16,17,18,19,20,21,22]. In the present series, we have analyzed both cerebral inflow and outflow; none of the patients showed cerebral inflow vascular problems, although outflow alterations were present in almost all patients (92%). To the best of our knowledge, this is the first report in the literature highlighting alterations of the extracranial venous circulation in recurrent BPPV.

Inner ear venous drainage occurs primarily through the internal auditory vein, the cochlear aqueduct vein, and the vestibular aqueduct vein. Consequently, the superior petrous sinus, the transverse sinus, and the superior bulb of the internal jugular vein represent the main outflow vessels. In particular, the vein of the vestibular aqueduct drains the venous flow of the utricle, the semicircular canals, the endolymphatic duct, and the sac [47]. Several theoretical models have been proposed in the literature to assess the pathogenetic link between venous inner ear alterations, inner ear metabolism, and homeostasis impairments. It is possible to speculate that vascular stasis due to a reduced cerebral outflow could affect specific sites of the inner ear, such as (i) the stria vascularis, actively involved in endolymph homeostasis, or (ii) the utricle and saccule maculae [48], with detrimental effects of the neuroepithelium and otolithic detachment. The persistence of a slow venous outflow, could eventually cause a venous flow inversion, leading to a portal-type circulation, in which venous capillary blood returns to the arterial capillary circulation, with detrimental consequences (i) to the inner ear neuroepithelium [49] and (ii) to the endolymphatic (changes in its glycosaminoglycans content have been postulated) [50].

The association between Ménière’s disease (MD) and CCSVI has been already reported [22,23]; it is likely that MD and recurrent BPPV could eventually retain a similar etiopathogenetic mechanism, involving inner ear microcirculation but at different sites [51,52,53,54]. In fact, it is possible to speculate that a slowed inner ear venous outflow could (i) induce a cytotoxic damage at the level of the stria vascularis [55], leading to MD; (ii) similarly, the same mechanism could affect the utricular macula, causing neuroepithelium damage and consequent otolithic detachment [46,56,57,58].

In the present study, 92% of patients with recurrent BPPV showed at least one ipsilateral (to the affected vestibule) outflow vascular alteration; in addition, 25% of the recruited patients had two vascular alterations (with at least one alteration ipsilateral to the affected vestibule). Moreover, 21% of patients reported three vascular alterations (with at least one alteration ipsilateral to the affected vestibule), including venous flow stenosis, blockages, and regurgitations or valvular abnormalities. The incidence of these ultrasound changes within the cerebral vascular outflow in recurrent BPPV subjects is significantly higher than that of the healthy population, which stands at approximately 11–12% [23].

Furthermore, it is known that jugular vein flow velocity values differ according to the head position in space. This feature is physiological because, while standing, the CSA of the jugular veins is reduced, and consequently, TAV tends to increase [8]. However, data from the present study show some ‘anti-physiological’ features. The first is that in the supine position, usually, moving towards the atrium, TAV values tend to increase from J3 to J2 and from J2 to J1. Interestingly, in BPPV subjects, TAV values increase, as expected, from J3 to J2, but then suddenly decelerate (J3 = 22.12 ± 10.81; J2 = 30.56 ± 15.97; J1 = 17.49 ± 10.90). Considering the physiological parameters collected in our previous publication, TAV values always increase from J3 to J2 and J2 to J1 (J3 = 22.2 ± 12.4; J2 = 30.1 ± 20.1; J1 = 40.8 ± 33.9) [8]. Furthermore, in the orthostatic position, TAV values of recurrent BPPV patients differ from those measured in healthy subjects, as per already published data (control J1 = 99.21 ± 45.06 vs. BPPV J1 = 59.70 ± 34.30) [8].

The present data corroborate the hypothesis that the hemodynamic alterations found in BPPV patients could be part of the still-unclear physio-pathological mechanisms of recurrent BPPV. A better understanding of the etiology and pathophysiological of recurrent BPPV is crucial. In fact, a clearer interpretation of the pathophysiological mechanisms of the vascular features of those affected by this disease is extremely important in order to achieve future eventual tailored treatments. The current recognized treatment for recurrent BPPV is represented by specific therapeutic maneuvers, according to the AAO–HNS criteria [1,24,29,35,36,37]. Pharmacological therapy can be proposed for the symptomatic treatment of BPPV, while repositioning maneuvers are the only effective therapeutic procedures (i.e., Epley or liberatory Semont maneuver and Gufoni or Barbecue roll maneuver for lateral canal BPPV) [24]. These results, if confirmed by larger and multicenter studies, may also introduce new treatment options for selected patients affected by recurrent BPPV (i.e., in case of persistent and recurrent cases), such as percutaneous transluminal angioplasty (PTA).

This is a pilot study and, as a consequence, presents some drawbacks. Major limitations of this study are (i) the small number of patients analyzed, (ii) the absence of a specific control group, and (iii) the fact that this investigation represents the experience of a single institution.

## 5. Conclusions

BPPV is one of the most common causes of peripheral vestibular pathology, and it can be very disabling in terms of quality-of-life perception, especially in its recurrent form.

Recurrent BPPV is more frequent in females, and its incidence increases with age.

Several studies in the literature have already reported that an impairment of inner ear microcirculation could represent a risk factor for recurrent BPPV; although preliminary, the data from the present study suggest that a chronic cerebral outflow impairment could also promote the onset of inner ear disorders, including recurrent BPPV. These anomalies (such as stenosis, blockages or regurgitations of flow, or abnormal valves, as per the CCSVI) could cause disruptions in venous inner ear drainage, hampering inner ear microcirculation and possibly causing recurrent otolith detachment.

Further investigations, including case–control and multi-center studies, are necessary to confirm this hypothesis.

## Figures and Tables

**Table 1:** Patient demographic data.

Sex	Age (Years)	Δt	Side	SC Involved
F	67	3	Right	PSC
F	51	48	Left	PSC
F	61	22	Left	PSC
F	55	16	Right	PSC
M	74	1	Right	PSC
F	53	3	Right	PSC
F	45	6	Left	PSC
F	63	12	Right	PSC
F	51	24	Right	PSC
M	60	31	Right	PSC
F	48	60	Right	PSC
F	59	60	Left	PSC
F	67	1	Right	LSC
M	68	60	Right	PSC
F	57	27	Right	PSC
F	59	4	Left	PSC
F	71	7	Right	LSC
F	66	24	Right	PSC
M	52	4	Right	LSC
F	42	2	Left	PSC
F	61	6	Left	PSC
F	62	3	Left	PSC
M	51	5	Right	LSC
F	62	5	Right	PSC

Abbreviation legend. M: male; F: female; Δt: interval in weeks between the first and second BPPV; SC: semicircular canal; PSC: posterior semicircular canal; LSC: lateral semicircular canal.

**Table 2:** Flow velocity of the major inflow and outflow routes of the brain within the recurrent BPPV study group.

Vascular Segment	TAVm (cm/s)			Peak Velocity (cm/s)		
	Ortho	Clino		Ortho	Clino	
	Mean ± sd	Mean ± sd	*p* Value	Mean ± sd	Mean ± sd	*p* Value
**CCA**	-	40.77 ± 8.97	-	-	-	-
**ICA**	-	32.86 ± 11.30	-	-	-	-
**ECA**	-	36.55 ± 7.82	-	-	-	-
**VA**	-	23.69 ± 10.35	-	-	-	-
**J3**	-	22.12 ± 10.81	-	-	-	-
**J2**	-	30.56 ± 15.97	-	-	-	-
**J1**	59.70 ± 34.30	17.49 ± 10.90	<0.0001	81.38 ± 44.37	46.27 ± 18.90	<0.0001
**VV**	19.77 ± 25.23	12.82 ± 19.24	0.0193	-	-	-

Abbreviation legend. TAVm: time average mean velocity; sd: standard deviation; CCA: common carotid artery; ICA: internal carotid artery; ECA: external carotid artery; VA: vertebral artery; J3: internal jugular vein in level 3; J2: internal jugular vein in level 2; J1: internal jugular vein in level 1; VV: vertebral vein.

## Data Availability

Not applicable.
